# Depression, anxiety and stress in women with breech pregnancy compared to women with cephalic presentation—a cross-sectional study

**DOI:** 10.1007/s00404-022-06509-0

**Published:** 2022-03-27

**Authors:** Madeleine Schauer, Elisabetta Latartara, Maria Alonso-Espias, Emma Rossetti, Pimrapat Gebert, Wolfgang Henrich, Larry Hinkson

**Affiliations:** 1 Department of Obstetrics, Charité Hospital, Humboldt University, Berlin, Germany; 2 Università Cattolica del Sacro Cuore Largo Francesco Vito, Rome, Italy; 3 La Paz University Hospital, Madrid, Spain; 4 University of Udine, Udine, Italy; 5 Institute of Biometry and Clinical Epidemiology, Charité–Universitätsmedizin Berlin, Corporate Member of Freie Universität Berlin, Humboldt-Universität Zu Berlin, Berlin Institute of Health, Berlin, Germany; 6 Berlin Institute of Health (BIH), Berlin, Germany; 7 8. Floor, Bettenhochhaus, Campus Mitte, Chariteplatz 1, 10117 Berlin, Germany

**Keywords:** Breech, Depression, Anxiety, Stress

## Abstract

**Purpose:**

This study aims to evaluate the level of psychological distress for women with breech compared to cephalic presentation. We hypothesized, that women with breech presentation have higher levels of depression, stress and anxiety. Secondary objectives were to analyze potential demographic risk factors and comorbidity of psychological distress in breech pregnancy.

**Methods:**

The breech study group was formed by 379 women with breech presentation. A sample of 128 women with cephalic presentation was recruited during routine clinical care. Depression, anxiety and stress symptoms were ascertained by means of the Depression–Anxiety–Stress-Score (DASS)-21 questionnaire. Categorial data was analyzed with Chi-square or exact test, continuous data with unpaired *t* test or Mann–Whitney *U* test. Demographic risk factors were identified using a binary logistic regression model.

**Results:**

Prevalence of psychological distress among women with breech was not higher compared to those of other pregnant women. Symptomatic depression, anxiety and stress affected 5.8%, 14.5% and 11.9% of women with breech, respectively. Decreasing age was identified as a risk factor for anxiety (*p* = 0.006). Multiparity increased risk for depression (*p* = 0.001), for anxiety (*p* = 0.026) and for perinatal stress (*p* = 0.010). More than 80% of women with depressive symptoms had comorbidities of psychological distress.

**Conclusions:**

Breech presentation compared to cephalic presentation was not associated with higher levels of psychological distress. However, breech pregnancies are affected by symptoms of potential mental disorder. Multiparous women and younger women may need additional support and would benefit from a standardized screening tool for the assessment of perinatal psychological distress.

**Clinical trial registration:**

Ethical approval (EA2/241/18) was granted by the Ethics Commission of the Charité University Hospital on the 23.01.2019 (ClinicalTrials.gov Identifier: NCT03827226).

**Supplementary Information:**

The online version contains supplementary material available at 10.1007/s00404-022-06509-0.

## Background

Approximately 2–4% of pregnancies are breech at term [1, 2]. Breech in itself is associated with higher stillbirth rate, fetal growth restriction and oligohydramnios [1]. In up to 95% of breech pregnancies a cesarean birth is planned [3–6]. Breech and its association with several adverse outcomes for mother and child represents a pregnancy with increased risk and this poses a challenge for the expectant mother [1].

The higher risk of perinatal complications can also be associated with psychological distress, such as pregnancy anxiety, which is defined as fear linked to the pregnancy itself (e.g. fears about oneself and baby’s well-being, concerns about labor and childbirth). Pregnancy anxiety is found to be one of the most potent risk factors for adverse outcomes. There is evidence, that conditions such as preterm birth are associated with pregnancy anxiety. Dunkel-Schetter et al. hypothesized, that medical risk conditions in the current pregnancy could contribute to this anxiety [7, 8]. In addition to high-risk-pregnancy other conditions such as increasing gestational age, younger maternal age and history of alcohol consumption are shown to be predictors for anxiety [9, 10].

Depression is also associated with high-risk pregnancy, lower maternal education and social factors, such as lack of social support and domestic violence [10–12].

Most expectant mothers generally have a desire for vaginal birth [13, 14]. However, when breech is diagnosed, women have to reconsider their mode of delivery based on risk. Elevated perinatal mortality, birth trauma, low APGAR scores and neonatal asphyxia are reported for vaginal breech compared to elective cesarean birth [3, 4, 15–21]. Cesarean birth minimizes the fetal risk, but increases risk for the mother and future pregnancies [15, 22–24]. One effective method to decrease cesarean birth for breech is the external cephalic version which is also an option. Fear of childbirth could occur due to the expectation of a complicated delivery fraught with risk. Wiklund et al. reported clinically significant fear of childbirth in 10% of women with cesarean section due to breech presentation [25].

For those women, who have a strong desire for vaginal delivery, it could be difficult to choose between the different options of dealing with their breech presentation. Firstly, they can decide on vaginal breech birth with its elevated perinatal risk. Secondly, they may have to withdraw from their expectation of a natural self-determined vaginal delivery and choose the cesarean section. Thirdly, they may decide on an attempt of external cephalic version, which, if successful, enables a vaginal cephalic delivery. The decision-making process in pregnancy thus has far-reaching consequences for both mother and child. Each decision may have a fundamental impact on further life, which poses an additional stressor to the mother. It is known, that pregnancy itself can be a stressful life event, and more so when a woman is confronted with the diagnosis of a medical complication or the risk of pregnancy loss [26]. The burden of decision-making in pregnancy plays a significant role and can potentiate the risk of developing depression, anxiety and stress [27, 28].

When confronted with a breech pregnancy, women may also be overburdened with decision-making before delivery with an increasing level of depression, anxiety and stress expected. Psychological distress in breech pregnancy has, however, never been investigated.

Psychological distress in pregnancy has been associated with outcomes, such as small for gestational age infants, lower birth weight and increased risk for preterm birth and postnatal depression [7, 8, 29–33]. Despite this, few units establish routine clinical screening for psychological distress in practice [12, 34, 35].

It has been shown that the prevalence of antenatal depression decreases from first to third trimester [29, 36, 37]. A cohort study found 13.5% of the participants at 32 weeks of gestation at risk for depression [38]. Other authors indicated a point prevalence between 8.5% and 11.1% for minor and major depression in the third trimester [29, 37, 39].

Indeed, antenatal anxiety is more prevalent than depression [9, 40, 41]. Lee et al. revealed that 54% of the women experienced symptoms of anxiety in at least one trimester and 35.8% in the last trimester [9].

The primary objective of this study was to evaluate the prevalence of symptoms of depression, anxiety, and stress for women with breech compared to women with cephalic presentation. We hypothesized higher levels of psychological distress among women with breech.

Secondary objectives were to determine (1) potential risk factors for high levels of distress among breech pregnancies, (2) the influence of gravidity on distress and (3) the level of comorbidity between depression, anxiety and stress.

## Methods

We conducted a prospective observational study between February 2019 and September 2020 in the obstetric clinic of Charité University Hospital Berlin.

Ethical approval (EA2/241/18) was granted by the Ethics Commission of the Charité University Hospital (ClinicalTrials.gov Identifier: NCT03827226). Written informed consent was provided by all women who agreed to take part in the study.

Clinical symptoms of depression, anxiety and stress were ascertained by means of the Depression Anxiety Stress Score (DASS)-21 questionnaire [42]. We recorded demographic data, including age, body mass index (BMI), gestational week, gravidity, parity, history of cesarean birth, spontaneous birth, miscarriage and pregnancy termination. Any pre-existing health-condition and gestational complication as well as smoking and consumption of alcohol or drugs were recorded.

The breech study group was recruited from pregnant women with breech presentation attending the consultant-led breech clinic. Breech was confirmed on sonographic examination from the 36th week of pregnancy. The consultation involved discussing the options of vaginal breech birth, attempting external cephalic version or planning a cesarean birth. We defined the following inclusion criteria: singleton gestation, age of at least 18 years, ability to sign the informed consent and basic German or English language skills. Exclusion criteria were history of mental disorder, use of antidepressant medication or anxiolytics and any fetal anomalies.

All women who met the inclusion criteria were asked to take part in the breech study group. A total of 409 women were initially included, six of them refused to continue. The response rate was 98.5%.

Recruitment of the control group was conducted by direct approach in the general obstetric outpatient clinic. We screened for eligibility among women who presented for normal delivery planning in the third trimester of pregnancy with cephalic presentation. Inclusion criteria were defined as singleton gestation, cephalic presentation, at least 30 weeks of gestation, age of at least 18 years, ability to sign the informed consent and basic German or English language skills. Exclusion criteria were the same as in the breech study group.

If a woman met all these requirements, she was informed about the study and asked to take part in the control study group. Information was either given by the study team or other health care workers. The response rate was 99.3%.

The DASS-21 is a self-report questionnaire consisting of 21 questions for measuring depression, anxiety and stress as negative emotional states. Developed by P. F. Lovibond and S. H. Lovibond as DASS-42, this short form version is also a well validated screening tool [42, 43].

The questionnaire consists of three 7-item subscales that measure depression, anxiety and stress. Patients estimate the degree of symptoms they have experienced over the last 7 days in a four-point-Likert-scale (0–3 points), with higher values indicating greater distress. The total score of each scale can range from 0 to 21 points and is built by summing all of the corresponding items. A total level of distress can be ascertained, ranging from 0 to 63.

Results can be converted in percentile ranks, which divide the scale in five severity groups, namely, normal (≤ 78), mild (79–87), moderate (88–95), severe (96–97) and extremely severe (≥ 98) [44]. Cutoff scores for symptomatic depression, anxiety and stress were defined as moderate level or greater, meaning scores of 7, 6 and 10, respectively.

The use of somatic items (e.g. fatigue, sleep disturbance, constipation and decreased appetite) in the assessment of depression is common [45, 46]. This can cause an overestimation of depressive symptoms in pregnancy. [47] Due to the absence of somatic items in DASS-21, it is more appropriate for screening in pregnancy.

### Statistical analysis

To calculate power we assumed the effect size of stress was 0.4, a sample of 379 in breech and 128 in control group, yielding a power of 97.4% with a significant level of 0.05 (two-sided) [48].

Continuous variables were presented with mean and standard deviation (SD), or median and interquartile range (25th percentile, 75th percentile), depending on the distribution. Histogram and Shapiro–Wilk test were used to explore the normal distribution. Chi-square or exact test by Monte-Carlo method was calculated for categorical data, whereas continuous data was analyzed with unpaired *t* test for normal distribution or Mann–Whitney *U* test for non-normal distribution.

To determine influencing factors for presence of psychological distress (moderate level or greater), we analyzed several potential demographic risk factors. Bivariate association between distress and demographic factors was carried out using Chi-square. Variables tested were: maternal age, BMI before pregnancy, week of gestation, history of breech, family history breech, pregnancy risk, gestational diabetes, pre-existing health condition, hypothyroidism, gravidity, parity, history of spontaneous birth, cesarean birth, miscarriage or pregnancy termination and fetal presentation. All variables that showed statistical significance, relevant factors (e.g. age, BMI) and variables, that differed significantly between breech and control group were included in a multiple logistic regression model. Presence or absence of depression, anxiety, stress and total distress were defined as dependent variables.

A *p* value < 0.05 was assumed as statistically significant. Data was analyzed with SPSS software version 25.0 (SPSS Inc., Chicago, Illinois, USA).

## Results

**Fig. 1:**
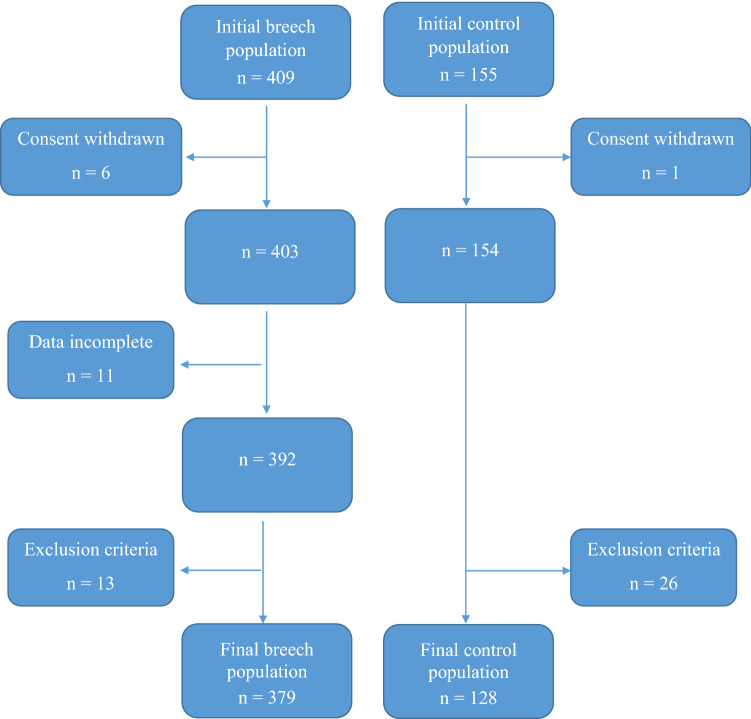
Patient recruitment process

A total of 564 women enrolled in the study, of which 57 women were excluded (Fig. 1). Of the remaining 507 pregnancies 379 had breech and 128 cephalic presentation.

Baseline characteristics group are shown in Table 1. There was no significant difference in maternal age and BMI between both groups (*p* = 0.070, *p* = 0.447).

**Table 1:** Baseline characteristics of breech and control study group

Variable	Breech (*n* = 379)	Control (*n* = 128)	
	*N* (%)	*N* (%)	*p* value
Maternal age			
Mean (SD)	32.66 (4.49)	33.49 (4.50)	0.070
< 20	1 (0.3)	1 (0.8)	0.451
20–29	81 (21.4)	21 (16.4)	
30–39	271 (71.4)	97 (75.8)	
≥ 40	26 (6.9)	9 (7.0)	
Body mass index (kg/m^2^)			
Mean (SD)	23.46 (4.11)	23.13 (4.79)	0.447
Underweight	19 (5.1)	11 (8.7)	0.410
Normal weight	250 (66.7)	84 (66.7)	
Overweight	79 (21.0)	21 (16.7)	
Obesity	27 (7.2)	10 (7.9)	
Week of gestation			
Mean (SD)	37.19 (0.79)	35.19 (2.30)	< 0.001
Gravidity			
Median (IQR)	1.00 (1.00, 2.00)	2.00 (1.00, 2.00)	0.009
Primigravida	231 (61.1)	63 (49.2)	0.018
Multigravida	147 (38.9)	65 (50.8)	
Parity			
0	283 (74.9)	74 (57.8)	0.001
1	72 (19.0)	38 (29.7)	
≥ 2	23 (6.1)	16 (12.5)	
History of miscarriage			
Spontaneous abortion	80 (21.2)	19 (15.0)	0.128
Pregnancy termination	13 (3.4)	12 (9.4)	0.007
History of delivery			
Vaginal	76 (20.1)	33 (25.8)	0.177
Cesarean birth	13 (3.4)	22 (17.3)	< 0.001
Gestational complications			
Diabetes	23 (6.1)	18 (14.2)	0.004
Others	13 (3.4)	17 (13.4)	< 0.001
Pre-existing health condition			
Hypothyroidism	46 (12.1)	24 (18.9)	0.056
Others	73 (19.3)	48 (37.8)	< 0.001

Table 2 shows median scores and prevalence of severity grades of the DASS-21 questionnaire. Clinical symptoms of moderate to severe depression symptoms were found in 5.8% of breech pregnancies. Symptomatic antenatal anxiety occurred in 14.5%. Symptomatic stress was found in 12%.

**Table 2:** Median (IQR) and prevalence of severity grades of the DASS-21 of breech (B) and control study group (C)

Variable	Breech = 379				Control = 128			
	Depression		Anxiety		Stress		Total	
	B	C	B	C	B	C	B	C
Score								
Median	1.00	1.00	2.00	2.50	4.00	4.00	7.00	8.00
(IQR)	(0.00, 2.00)	(0.00, 2.75)	(1.00, 4.00)	(1.00, 5.00)	(2.00, 7.00)	(2.00, 7.00)	(4.00, 13.00)	(4.00, 13.75)
* p* value	0.577		0.035		0.597		0.404	
Severity grades *N* (%)								
Normal	338 (89.2)	113 (88.3)	279 (73.6)	81 (63.3)	300 (79.1)	103 (80.4)	292 (77.0)	96 (75.0)
Mild	19 (5.0)	6 (4.7)	45 (11.9)	19 (14.8)	34 (9.0)	13 (10.2)	37 (9.8)	14 (10.9)
Moderate	14 (3.7)	7 (5.4)	38 (10.0)	16 (12.5)	27 (7.1)	10 (7.8)	44 (11.6)	16 (12.5)
Severe	5 (1.3)	1 (0.8)	10 (2.6)	6 (4.7)	12 (3.2)	1 (0.8)	3 (0.8)	1 (0.8)
Extremely severe	3 (0.8)	1 (0.8)	7 (1.9)	6 (4.7)	6 (1.6)	1 (0.8)	3 (0.8)	1 (0.8)
*p* value	0.904		0.120		0.660		0.976	

**Fig. 2:**
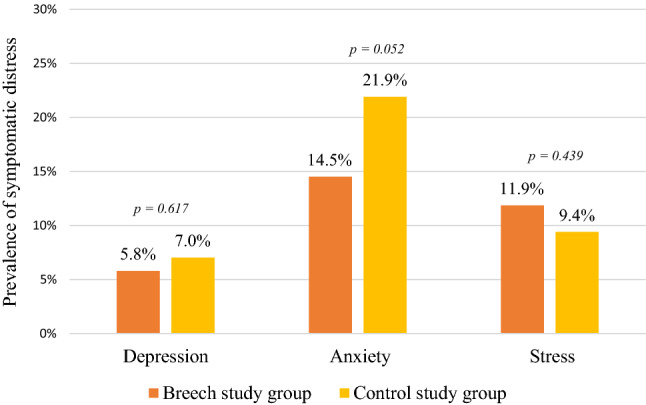
Prevalence of symptomatic distress (moderate to extremely severe) measured by the DASS-21 for breech and control study group

Prevalence of symptomatic psychological distress (moderate to extremely severe) is presented in Fig. 2.

A Mann–Whitney *U* test showed with no significant differences for scores of depression, stress and total distress between the groups. The median anxiety score of the control study group was significantly higher (*p* = 0.033).

No significant results were found for differences in severity grades, neither for the recommended five grades (normal to extremely severe) nor for those defined by our team (normal and symptomatic).

To determine demographic confounders and the influence of the fetal presentation on the presence of symptoms of mental disorders, a multiple logistic regression model was performed (Table 3).

**Table 3:** Binary logistic regression model for demographic confounders of distress

Variables	All pregnancies (*n* = 507)							
	Depression		Anxiety		Stress		Total	
	aOR (95% CI)	*p* value	aOR (95% CI)	*p* value	aOR (95% CI)	*p* value	aOR (95% CI)	*p* value
Maternal age (yr)	0.98 (0.89–1.07)	0.657	0.93 (0.88–0.99)	0.019	0.95 (0.89–1.02)	0.150	0.95 (0.89–1.01)	0.078
BMI (kg/m^2^)	1.06 (0.98–1.14)	0.150	1.03 (0.98–1.08)	0.303	1.03 (0.97–1.10)	0.360	1.02 (0.97–1.09)	0.417
Week of gestation	0.94 (0.72–1.23)	0.653	0.986 (0.84–1.16)	0.870	0.79 (0.63–1.01)	0.059	1.01 (0.84–1.22)	0.934
Parity								
0	1.00	–	1.00	–	1.00	–	1.00	–
1	1.69 (0.61–4.66)	0.309	1.18 (0.62–2.27)	0.613	1.55 (0.74–3.29)	0.247	1.38 (0.69–2.77)	0.367
≥ 2	7.63 (2.66–21.84)	< 0.001	2.52 (1.07–5.95)	0.035	4.73 (1.88–11.93)	0.001	3.93 (1.65–9.33)	0.002
History of cesarean section								
Yes vs. no	1.06 (0.28–4.05)	0.927	0.54 (0.18–1.67)	0.287	0.92 (0.29–2.96)	0.894	0.92 (0.32–2.69)	0.882
History of pregnancy termination								
Yes vs. no	1.01 (0.20–5.07)	0.994	1.71 (0.65–4.48)	0.277	0.90 (0.24–3.37)	0.880	1.44 (0.49–4.23)	0.502
Gestational diabetes								
Yes vs. no	0.56 (0.12–2.64)	0.459	1.50 (0.68–3.31)	0.316	2.23 (0.93–5.36)	0.073	2.22 (1.00–4.96)	0.051
Gestational complications								
Yes vs. no	1.17 (0.25–5.57)	0.845	1.63 (0.64–4.16)	0.307	1.39 (0.43–4.49)	0.578	1.41 (0.49–4.09)	0.527
Hypothyroidism								
Yes vs. no	0.98 (0.32–3.04)	0.975	1.18 (0.59–2.37)	0.646	0.60 (0.22–1.63)	0.319	1.27 (0.60–2.70)	0.535
Other pre-existing health condition								
Yes vs. no	1.24 (0.51–3.02)	0.638	1.85 (1.07–3.19)	0.027	1.03 (0.51–2.08)	0.944	1.55 (0.84–2.85)	0.158
Fetal presentation								
Cephalic	1.00	–	1.00	–	1.00	–	1.00	–
Breech	1.10 (0.38–3.22)	0.861	0.73 (0.38–1.38)	0.327	2.59 (0.95–7.04)	0.063	1.19 (0.57–2.49)	0.649
Nagelkerke *R*^2^ (%)		9.7		7.0		8.7		6.8
Hosmer and Lemeshow test (*p* value)		0.658		0.053		0.109		0.677

Linearity was assessed using the Box–Tidwell procedure. [49] All variables were found to follow a linear relationship. Multicollinearity was checked if the correlation coefficient between independent variables is higher than 0.7. [50] Collinearity was found between gravidity and parity variable (*r* = 0.711); therefore, gravidity was not included into the model.

Goodness-of-fit was assessed using the Hosmer–Lemeshow test, indicating a good model fit for all dependent variables, (depression: χ^2^ (8) = 5.91, *p* = 0.658; anxiety: χ^2^ (8) = 15.34, *p* = 0.053; stress: χ^2^ (8) = 13.08, *p* = 0.109; total: χ^2^ (8) = 5.74, *p* = 0.677). The model is resulting in a low amount of explained variance [51], as shown by Nagelkerke’s *R*^2^ (depression: *R*^2^ = 0.097; anxiety: *R*^2^ = 0.070; stress: *R*^2^ = 0.087; total: *R*^2^ = 0.068).

Preexisting health condition (hypothyroidism excluded) had a 1.85 times higher risk of anxiety (*p* = 0.027). Multiparity significantly increased risk for depression, anxiety, stress and total distress.

After adjusting for age, BMI as well as gestational and medical history, fetal presentation was not a significant predictor of psychological distress in pregnancy. Breech pregnancies were 2.59 times more likely to be stressed during third trimester (*p* = 0.063), but had lower risk for anxiety; however, these results were not statistically significant.

A multiple logistic regression model was performed to determine risk factors for psychological distress with breech. Adjusted Odds Ratios (95% CI) are shown in Table 4.

**Table 4:** Multiple logistic regression model for risk factors of distress in breech pregnancy

Variables	Depression		Anxiety		Stress		Total	
	aOR (95% CI)	*p* value	aOR (95% CI)	*p* value	aOR (95% CI)	*p* value	aOR (95% CI)	*p* value
Maternal age (yr)	1.01 (0.91–1.13)	0.824	0.91 (0.85–0.97)	0.006	0.95 (0.88–1.03)	0.212	0.95 (0.89–1.02)	0.186
Body mass index								
Underweight	0.64 (0.07–6.17)	0.701	1.86 (0.55–6.32)	0.322	1.55 (0.40–6.10)	0.529	1.22 (0.31–4.76)	0.777
Normal weight	1.00	–	1.00	–	1.00	–	1.00	–
Overweight	1.00 (0.30–3.33)	0.996	1.41 (0.67–2.96)	0.367	0.69 (0.28–1.75)	0.438	0.82 (0.35–1.88)	0.631
Obesity	2.71 (0.73–10.04)	0.136	2.30 (0.87–6.08)	0.095	2.20 (0.79–6.10)	0.131	1.91 (0.69–5.29)	0.211
Parity								
0	1.00	–	1.00	–	1.00	–	1.00	–
1	1.35 (0.41–4.48)	0.625	1.16 (0.54–2.52)	0.705	1.55 (0.68–3.50)	0.296	1.24 (0.56–2.76)	0.597
≥ 2	8.61 (2.50–29.57)	0.001	3.35 (1.15–9.74)	0.026	4.27 (1.42–12.86)	0.010	4.33 (1.53–12.27)	0.006
Gestational diabetes								
Yes vs. no	0.37 (0.04–3.72)	0.398	2.08 (0.75–5.76)	0.157	2.61 (0.87–7.85)	0.088	2.83 (1.00–7.99)	0.050
Gestational complications								
Yes vs. no	1.48 (0.17–12.85)	0.725	1.21 (0.25–6.01)	0.814	1.66 (0.34–8.13)	0.533	1.35 (0.27–6.66)	0.713
Hypothyroidism								
Yes vs. no	0.65 (0.14–3.12)	0.589	0.74 (0.27–2.05)	0.564	0.52 (0.15–1.83)	0.307	0.83 (0.30–2.31)	0.727
Other pre-existing health condition								
Yes vs. no	1.27 (0.42–3.89)	0.675	1.75 (0.85–3.60)	0.132	1.24 (0.55–2.81)	0.607	1.43 (0.67–3.06)	0.361

Multiparity was found to be the most important risk factor for symptoms of mental disorder. Women who have given birth to at least two children had an eightfold higher risk for depression (*p* = 0.001), threefold higher risk for anxiety (*p* = 0.026) and fourfold higher risk for stress (*p* = 0.010) compared to nulliparous women. Being younger increased the risk for the presence of symptomatic anxiety (*p* = 0.006). Gestational diabetes was 2.83 times more likely to cause high total distress scores (*p* = 0.050) and 2.61 times increased risk for stress, although this association was not significant (*p* = 0.088).

Breech pregnancies were divided into four groups: (1) primigravida, (2) multigravida with either history of term delivery, (3) history of miscarriage or (4) history of delivery and miscarriage.

Multigravida compared to primigravida were significantly more stressed (*p* = 0.046) and had higher total scores (*p* = 0.044).

Another relation was found for primigravida vs. multigravida with history of term delivery. Multigravida had higher depression scores (*p* = 0.007) as well as stress scores (*p* = 0.043).

All other combinations, especially the comparison between history of delivery and history of miscarriage did not show significant results. Comorbidity with depression and anxiety occurred in 3.6%, depression and stress in 4.2% and anxiety and stress in 6.3% of breech cases.

**Fig. 3:**
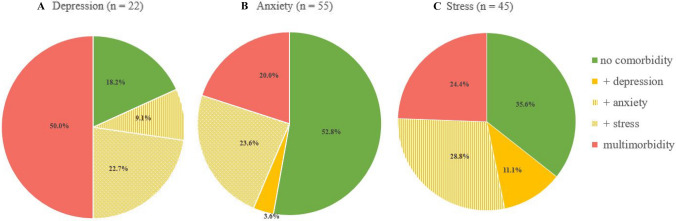
Psychological comorbidities of women with breech presentation. Subgroup analysis of women with symptoms of depression (**A**), anxiety (**B**) and stress (**C**). They had either no comorbidity (green: only one scale of the DASS-21 above cutoff), one comorbidity (yellow: two scales above cutoff) or multimorbidity (red: all three scales above cutoff)

Multimorbidity (symptoms of depression, anxiety and stress simultaneously) was found in 11 women, representing 50.0%, 20.0% and 24.4% of the depressive, anxious and stressed population, respectively (Fig. 3). Of those with symptomatic depression, 9.1% were comorbid for anxiety and 22.7% for stress, the other 18.2% had no comorbid distress. Of those women with symptoms of anxiety, more than half had no comorbid distress, another 3.6% rated themselves as depressive and 23.6% as stressed. Combinations with either depression or anxiety was found in 11.1% and 28.8% of the participants suffering from stress, whereas a third of the stressed women had no other psychological comorbidities.

## Discussion

### Main findings

The prevalence of symptoms for moderate to extremely severe depression, anxiety and stress in the breech pregnancy was 5.8%, 14.5% and 11.9%, respectively. There was no statistical difference compared to cephalic pregnancy.

### Interpretation

Studies show that women with high-risk pregnancy have a higher risk of psychological distress in pregnancy [10, 52]. We used the well-established DASS-21 questionnaire to assess symptoms of depression, anxiety and stress in breech compared to cephalic pregnancy [42, 43].

In the breech group symptoms of mild depression were present in 5% and moderate to extremely severe symptoms in 5.8%. One study by Barber et al. found 10.5% of pregnant women as mildly depressive and 21.5% as moderate or highly depressive [48]. In this study, however, women with known mental disorders were not excluded, pregnancies with breech were not defined and an online survey was used. Other studies showed variable prevalence rates of 13.5% and 11.1% for depression in pregnancy with the Edinburgh Postnatal Depression Scale [29, 38]. These variations are likely because women with known mental disorders were not excluded, and gestational ages were also not defined. A meta-analysis on prevalence and incidence of perinatal depression excluded studies based on self-report screens and found a point prevalence of 8.5% for minor and major depression [39].

Mild and symptomatic anxiety was found in 11.9% and 14.5% of the breech study group. Other studies such as Barber et al. and Lee et al. showed higher rates (31.2% and 35.8%) for symptomatic anxiety in pregnancy [9], 48. However, these studies were limited as patients with known mental disorders were not excluded, gestational age and fetal presentation was not defined and different screening tools were used. A meta-analysis by Dennis et al. could identify the difference between the prevalence of anxiety over all trimesters, when ascertained by self-report symptoms (18.0%) or clinical diagnosis as a measurement (15.2%) [40]. Prevalence in third trimester was determined as being 24.6% for self-report and 15.4% for clinical diagnosis of any anxiety disorder, similar to the incidence in our breech study group.

In our study, 9% of breech pregnancies suffered from mild stress and 11.9% from moderate to extremely severe stress. Compared to depression and anxiety, antenatal stress seems to be neglected in research as it does not offer a medical diagnosis of a mental disorder. Two studies on stress in pregnancy presented varying results. Woods et al. used a clinical screening protocol for psychosocial strain and found 78% had low to moderate and 6% high stress levels [34]. In comparison, Barber et al. found that 16.3% of pregnant women had moderate or higher levels of stress [48].

We performed an analysis of prevalence rates of combined components of psychological disorders in the breech population. Comorbid depression and anxiety affected 3.6%, depression and stress 4.2% and anxiety and stress 6.3%. Of the women with symptoms of depression, we found more than 80% had high levels of other psychological comorbidities. Comorbidity of antenatal depression and anxiety has been investigated in numerous studies [9, 31, 41, 53, 54]. A meta-analysis showed in the third trimester that comorbid anxiety and mild to severe depression occurred in 9.5% of all cases and moderate to severe depression in 6.6% [54]. There remains a paucity in the literature, however, on the role of mental stress in pregnancy; therefore, the association with other comorbidities has been rarely described.

We also analyzed predictors of psychological disorder in women with breech. We found that women with multiparity, gestational diabetes and decreasing age were at higher risk. In the literature there are conflicting results on the influence of parity. Fairbrother et al. found no significant difference between nulliparas and multiparas, Dipietro et al. found a higher prevalence among multiparas, whereas Gillespie et al. found a higher prevalence in primiparas [55–57].

We found that breech pregnancy beyond 36 weeks of pregnancy, even with the challenges of deciding on interventions did not have significantly higher levels of psychological distress symptoms, compared to cephalic pregnancies. In fact, women with cephalic presentation in the control group scored higher on the anxiety subscale. Presumably, this might be a result of the collocation of the control group with other obstetric complications (e.g. hypertension, gestational diabetes, oligohydramnios, previous cesarean birth, previous stillbirth) which are associated with higher rates of anxiety [10]. However, a logistic regression analysis of our study and control groups showed no influence of these comorbidities on the presence of psychological distress. Anxiety could also be affected by fear of childbirth in general. Laursen et al. observed a significant association of fear of childbirth with depressive and anxious symptoms [58]. Rouhe et al. found that fear of childbirth affects primarily nulliparous women and women with a history of cesarean birth [59]. A proportion of women presenting in the breech clinic may perhaps be more desirous for the chance to deliver vaginally and may have less fear of childbirth.

### Clinical implications

Whilst we initially hypothesized that the burden of decision-making in the presence of a breech presentation beyond the 36 weeks of gestation presents an additional risk for the development of psychological distress in pregnancy, we could not prove this hypothesis and it appears that women with a breech pregnancy generally speaking, are exposed to the same prevalence of depression anxiety and stress as women with non-breech pregnancies. Importantly, however, special attention needs to be reserved for high-risk groups which we identified, such as multiparous mothers, gestational diabetes, and younger mothers, where we found significantly higher rates of anxiety. These especially high-risk groups warrant additional psychological and social support to benefit mother and child. We also found the general incidence of depression, anxiety, and stress in pregnancy enough to recommend and warrant in clinical practice the routine implementation of a screening program for psychological problems in pregnancy.

### Strengths and limitations

This is a large prospective study performed on over 370 participants with breech. This is the first study looking at breech and the influence of perinatal psychological distress symptoms. Importantly, all women with pre-known mental disorders were excluded to minimize selection bias and a well validated screening tool was used [43].

The DASS-21 self-report questionnaire is, however, time dependent, evaluating symptom over the past week. Negative emotional states at other timepoints may have influenced self-assessment scores. Nevertheless, questionnaires are not used for diagnosis, but rather as a screening tool. Socio-economic confounders such as relationship status were not ascertained. There is already extensive research done on social predictors of psychological distress and we chose to look primarily at clinical parameters.

Despite no increase in psychological distress symptoms in women with breech in general, we found that those associated with multiparity, gestational diabetes and decreasing age are at higher risk of developing a mental illness and, therefore, require additional support. Screening for mental disorders should be established in clinical routine to detect women who may be at high risk of mental illness. Hare et al. recently showed that anxiety in pregnancy was associated with a 15% increased risk of postnatal depression and this can negatively influence mother–infant bonding [60]. Optimal antenatal care should, therefore, include assessment of the mental health status of expecting mothers and screening for stress in particular. Furthermore, perinatal and delivery complications such as can occur with breech pregnancy can increase a child’s risk for anxiety independent from the parental psychopathology [61]. Freed et al. also found it useful to screen for anxiety in pregnancy especially in mothers with a known psychiatric disease, such as bipolar disorder, as this can influence psychopathology in offspring [62].

Support should then be tailored to meet individual needs. We, therefore, recommend a standardized screening tool such as the DASS-21 questionnaire for the specific categories of perinatal psychological distress to make comparisons between future studies compatible. Indeed, prenatal screening for anxiety can be implemented into prediction models used to earlier identify mothers and offspring at risk [63, 64].

## Conclusions

In women with breech pregnancies significant symptomatic depression, anxiety and stress symptoms were found in 5.8%, 14.5% and 11.9%, respectively. Compared to cephalic pregnancy this was not higher. However, multiparity, gestational diabetes and decreasing maternal age were identified as potential factors for developing mental distress in breech pregnancies and require additional support.

## Supplementary Information

Below is the link to the electronic supplementary material.

Supplementary file1 (DOCX 19 KB)

Supplementary file2 (DOC 100 KB)

## Data Availability

Data sharing is available on reasonable request from the corresponding author.

## Abbreviations

ECV: External cephalic version
DASS-21: Depression, anxiety, stress score-21
BMI: Body mass index
