# Supplementary material for: Depression, anxiety and stress in women with breech pregnancy compared to women with cephalic presentation—a cross-sectional study
Source: Arch Gynecol Obstet. 2022 Mar 27;307(2):409–19. doi: 10.1007/s00404-022-06509-0 (PMC9918572; doi:10.1007/s00404-022-06509-0)
Supplement: Supplementary file 1 — Supplementary file1 (DOCX 19 KB) [file 404_2022_6509_MOESM1_ESM.docx]

**Fragen zu Ihrem Befinden**

*Bearbeitungshinweis:* Bitte lesen Sie jede Aussage und kreuzen Sie die Zahl 0, 1, 2 oder 3 an, die angeben soll, wie sehr die Aussage **während der letzten Woche** auf Sie zutraf. Es gibt keine richtigen oder falschen Antworten. Versuchen Sie, sich spontan für eine Antwort zu entscheiden.

0 Traf **gar nicht** auf mich zu

1 Traf **bis zu einem gewissen Grad** auf mich zu oder **manchmal**

2 Traf **in beträchtlichem Maße** auf mich zu oder **ziemlich oft**

3 Traf **sehr stark** auf mich zu oder **die meiste Zeit**

| 1 Ich fand es schwer, mich zu beruhigen. | 0 | 1 | 2 | 3 |
| --- | --- | --- | --- | --- |
| 2 Ich spürte, dass mein Mund trocken war. | 0 | 1 | 2 | 3 |
| 3 Ich konnte überhaupt keine positiven Gefühle mehr erleben | 0 | 1 | 2 | 3 |
| 4 Ich hatte Atemprobleme (z.B. übermäßig schnelles Atmen, Atemlosigkeit ohne körperliche Anstrengung). | 0 | 1 | 2 | 3 |
| 5 Es fiel mir schwer, mich dazu aufzuraffen, Dinge zu erledigen. | 0 | 1 | 2 | 3 |
| 6 Ich tendierte dazu, auf Situationen überzureagieren. | 0 | 1 | 2 | 3 |
| 7 Ich zitterte (z.B. an den Händen). | 0 | 1 | 2 | 3 |
| 8 Ich fand alles anstrengend. | 0 | 1 | 2 | 3 |
| 9 Ich machte mir Sorgen über Situationen, in denen ich in Panik geraten und mich lächerlich machen könnte. | 0 | 1 | 2 | 3 |
| 10 Ich hatte das Gefühl, dass ich mich auf nichts mehr freuen konnte. | 0 | 1 | 2 | 3 |
| 11 Ich bemerkte, dass ich mich schnell aufregte. | 0 | 1 | 2 | 3 |
| 12 Ich fand es schwierig, mich zu entspannen. | 0 | 1 | 2 | 3 |
| 13 Ich fühlte mich niedergeschlagen und traurig. | 0 | 1 | 2 | 3 |
| 14 Ich reagierte ungehalten auf alles, was mich davon abhielt, meine momentane Tätigkeit fortzuführen. | 0 | 1 | 2 | 3 |
| 15 Ich fühlte mich einer Panik nahe. | 0 | 1 | 2 | 3 |
| 16 Ich war nicht in der Lage, mich für irgendetwas zu begeistern. | 0 | 1 | 2 | 3 |
| 17 Ich fühlte mich als Person nicht viel wert. | 0 | 1 | 2 | 3 |
| 18 Ich fand mich ziemlich empfindlich. | 0 | 1 | 2 | 3 |
| 19 Ich habe meinen Herzschlag gespürt, ohne dass ich mich körperlich angestrengt hatte (z.B. Gefühl von Herzrasen oder Herzstolpern). | 0 | 1 | 2 | 3 |
| 20 Ich fühlte mich grundlos ängstlich. | 0 | 1 | 2 | 3 |
| 21 Ich empfand das Leben als sinnlos. | 0 | 1 | 2 | 3 |

DASS21-G Nilges, Essau, 2015[1]

1. Nilges P, Essau C: **[Depression, anxiety and stress scales: DASS--A screening procedure not only for pain patients]**. *Schmerz* 2015, **29**(6):649-657.

Website access to the DASS-21 Questionnaire in different languages: http://www2.psy.unsw.edu.au/dass
